# General and species-specific transcriptional responses to downy mildew infection in a susceptible (*Vitis vinifera*) and a resistant (*V. riparia*) grapevine species

**DOI:** 10.1186/1471-2164-11-117

**Published:** 2010-02-18

**Authors:** Marianna Polesani, Luisa Bortesi, Alberto Ferrarini, Anita Zamboni, Marianna Fasoli, Claudia Zadra, Arianna Lovato, Mario Pezzotti, Massimo Delledonne, Annalisa Polverari

**Affiliations:** 1 Department of Biotechnology, University of Verona, Strada le Grazie 15, 37134 Verona, Italy; 2 Department of Agricultural and Environmental Science, University of Perugia, B.go XX Giugno 72, 06121 Perugia, Italy

## Abstract

**Background:**

Downy mildew is a destructive grapevine disease caused by *Plasmopara viticola *(Berk. and Curt.) Berl. and de Toni, which can only be controlled by intensive fungicide treatments. Natural sources of resistance from wild grapevine (*Vitis*) species are used in conventional breeding approaches, but the signals and effectors involved in resistance in this important crop species are not well understood.

**Results:**

Early transcriptional changes associated with *P. viticola *infection in susceptible *V. vinifera *and resistant *V. riparia *plants were analyzed using the Combimatrix microarray platform. Transcript levels were measured 12 and 24 h post-inoculation, reflecting the time points immediately preceding the onset of resistance in *V. riparia*, as determined by microscopic analysis. Our data indicate that resistance in *V. riparia *is induced after infection, and is not based on differences in basal gene expression between the two species. The strong and rapid transcriptional reprogramming involves the induction of pathogenesis-related proteins and enzymes required for the synthesis of phenylpropanoid-derived compounds, many of which are also induced, albeit to a lesser extent, in *V. vinifera*. More interestingly, resistance in *V. riparia *also involves the specific modulation of numerous transcripts encoding components of signal transduction cascades, hypersensitive reaction markers and genes involved in jasmonate biosynthesis. The limited transcriptional modulation in *V. vinifera *represents a weak attempted defense response rather than the activation of compatibility-specific pathways.

**Conclusions:**

Several candidate resistance genes were identified that could be exploited in future biotechnological approaches to increase disease resistance in susceptible grapevine species. Measurements of jasmonic acid and methyl jasmonate in infected leaves suggest that this hormone may also be involved in *V. riparia *resistance to *P. viticola*.

## Background

*Plasmopara viticola *(Berk. and Curt.) Berl. and de Toni is an oomycete pathogen that causes downy mildew in grapevine. This devastating disease causes partial or total crop losses and has a severe secondary environmental impact due to the repeated fungicide applications required as a control measure. *P. viticola *is an obligate pathogen that obtains nutrients from infected plant cells through specialized structures known as haustoria, which also allow the exchange of signals involved in the establishment of compatibility [1]. In susceptible grapevine genotypes, compatibility is probably achieved through a lack of recognition. Some oomycetes can secrete effectors that suppress host cell defense responses but such effectors have yet to be described in *P. viticola *[2,3].

Although European *V. vinifera *cultivars are highly susceptible to *P. viticola*, *Muscadinia *species and several American and Asian *Vitis *species exhibit varying levels of resistance, allowing quantitative trait loci (QTLs) and major resistance genes to be mapped [4-9]. Efforts to introgress these traits into cultivated *V. vinifera *genotypes by conventional breeding have produced some resistant interspecific hybrids, but further work is needed to couple strong resistance with high quality wine production [10]. This process will be greatly accelerated by the availability of the grapevine genome sequence [11,12] and high density genetic maps [13,14].

Detailed resistance mechanisms have been described in a few model species [15], and these often involve a signal transduction cascade triggered by infection which induces the resistance response. Plants can recognize general elicitors (or pathogen-associated molecular patterns, PAMPs) and specific elicitors encoded by pathogen *Avr *genes, as well as byproducts of pathogen activity (damage-associated molecular patterns, DAMPs), through a wide repertoire of receptors, with intriguing similarity to the innate immune system in animals [16,17]. Defense responses include strengthening the cell walls [18], the synthesis of pathogenesis-related (PR) proteins and antimicrobial compounds such as phytoalexins [19], and the hypersensitive response (HR), in which cells undergo programmed cell death in the infected region to block further spreading of the pathogen [20].

Wild American grapevine species may enjoy a higher level of constitutive resistance to *P. viticola *because of the higher basal level of certain antimicrobial compounds [21-25]. Post-infection resistance mechanisms have also been described in wild *Vitis *species, including the accumulation of reactive oxygen species, PR proteins, antimicrobial compounds, peroxidases and HR activation [26-31]. Although *V. vinifera *is susceptible to *P. viticola*, it can defend itself against other pathogens indicating the defense components are in place but are not activated in response to this pathogen [28]. The early signaling events underlying defense responses in grapevine have only recently been described [32-37] but a systematic survey of the *V. vinifera *genome has identified more than 200 resistance gene analogs, many localized in genomic regions associated with *P. viticola *resistance in wild *Vitis *spp. [12,38], as well as orthologs of Arabidopsis genes that regulate defense pathways [39,40].

In this paper we describe the early transcriptional changes associated with *P. viticola *infection in both susceptible *Vitis vinifera *and resistant *Vitis riparia *plants, performed on a Combimatrix Grapevine Microarray, the broadest transcriptomics resource available for *Vitis *species http://www.combimatrix.com/tech_microarrays.htm. Transcriptomic approaches have been used to analyze plant-pathogen interactions in model species. Although several grapevine diseases have been investigated using Affymetrix [23,36,37] or Operon grapevine chips [33], *P. viticola *is not among them. Our study therefore provides the first broad overview of the molecular events underlying the early response to *P. viticola *infection in susceptible and resistant grapevine species and will provide valuable candidate genes that could be used to develop mildew-resistant commercial grapevine plants.

## Results

### *P. viticola *developmental stages

**Figure 1:**
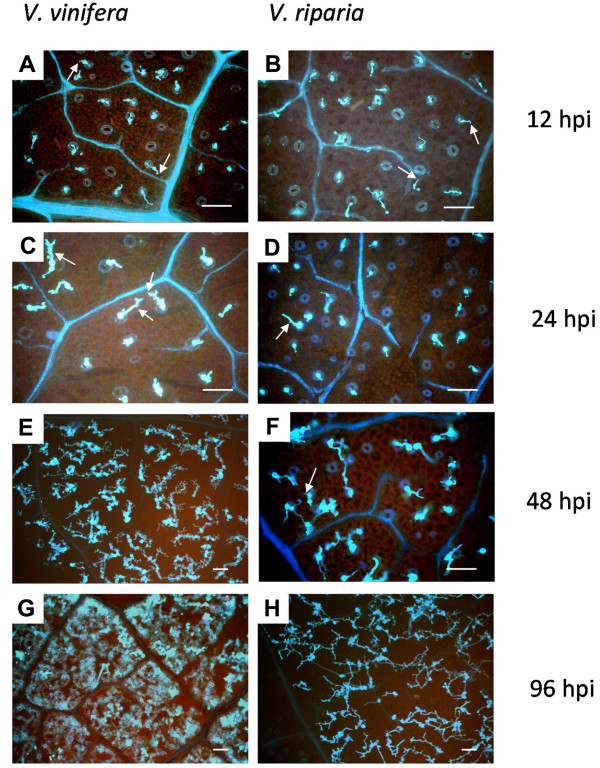
**Analysis of *P. viticola *infection steps**. Infected leaf disks from *V. vinifera *(left panels) and *V. riparia *(right panels) were collected at 12, 24, 48 and 96 hpi, stained with 0.05% aniline blue and observed under an epifluorescence microscope. Panels **A**, **B**, **C**, **D **and **F**: magnification 200×; panels **E**, **G **and **H**: magnification 100×. Arrows indicate primary hyphae, arrowheads haustoria. Bars = 80 μm.

After inoculating plants with *P. viticola*, we followed the progress of the infection by looking at the developmental time-course of the pathogen. On that basis we chose which RNA samples were most suitable for microarray analysis. Leaf samples were collected at 12, 24, 48 and 96 hours post-inoculation (hpi) and stained with aniline blue for microscopy (Figure 1). Zoospores were localized over stomata by 12 hpi in both species, and germ tubes, primary hyphae and the first haustoria could be identified. By 24 hpi, further mycelium development appeared to be delayed in *V. riparia*. By 48 hpi, a mycelium network with many haustoria was observed in *V. vinifera*, whereas branched hyphae with only a few haustoria were observed in *V. riparia*. At 96 hpi, *V. vinifera *tissues were completely invaded by mycelia and heavy sporulation followed, whereas only small patches of mycelium were visible in *V. riparia*, and sporulation was severely impaired or absent. This established that the resistance response in *V. riparia *probably began within the first 24 hpi, and we therefore chose 12 and 24 hpi as the relevant time-points for microarray analysis.

### Reliability of hybridization data

Both phylogenetic analysis [41,42] and previous cross-species microarray analysis using *Vitis *species [22] suggested that a *V. vinifera *microarray should reliably detect transcriptional changes in *V. riparia*. However, a certain level of sequence divergence between the two species could increase the random noise in the hybridization data and possibly result in a lower correlation between *V. riparia *replicates compared to *V. vinifera *replicates. We tested healthy samples collected at 12 and 24 hpi, which served as controls in the infection experiments, and found no evidence for differences in the correlation between replicates for each species. The average Spearman's rank correlation coefficient (r) between *V. vinifera *replicates was 0.9678 (range 0.9536-0.9782), which was comparable to the *V. riparia *replicates (r = 0.9349; range 0.9017-0.9680).

We also checked the intensity distribution of log2-transformed data, the overall hybridization intensity and the number of absent calls (i.e. transcripts with a fluorescence signal below a calculated threshold, see Materials and Methods) for the two species. The intensity distributions of data derived from uninfected samples of each species were normal-like and similar. The average log2-transformed abundance values were 8.57 ± 2.07 and 7.40 ± 2.74 (across-replicate average ± SD) in *V. riparia *and *V. vinifera*, respectively. The number of probe sets assigned an absent call was 7,712 in *V. riparia*, and 7,306 in *V. vinifera*. These observations confirmed the reliability and comparability of the microarray results in the two grapevine species.

### Interspecies differences in basal gene expression

Differences in basal gene expression between the two grapevine species were determined by comparing matched uninfected control samples for the steady-state levels of all 24,571 transcripts represented on the microarray. However, because it has been suggested that resistance in *V. riparia *could in part reflect constitutive physical or chemical barriers, we also focused on defense-related transcripts (i.e. those functionally associated with disease resistance, stress, the cell wall and secondary metabolism). Because the 12 hpi samples were harvested in darkness and the 24 hpi samples in daylight, data from the different time-points were normalized and compared separately to avoid the detection of genes regulated by light. We identified 5550 and 6379 transcripts with statistically significant differential expression at 12 and 24 hpi, respectively (Additional files 1 and 2). At both time points, ~ 48% of the differentially expressed transcripts were more abundant in *V. riparia *and ~ 52% were more abundant in *V. vinifera*. Broadly similar results were obtained when restricting the analysis to defense-related transcripts. Here ~ 45% of the differentially expressed transcripts were more abundant in *V. riparia *and ~ 55% were more abundant in *V. vinifera *(Additional files 1, 2 and 3).

To exclude genes regulated by light in only one of the species, we also retrieved the subset of 2176 transcripts present at both time points (Additional file 4). In this group, many transcripts were more abundant in one species at one time point but more abundant in the other species at the other time point, and there was a trend showing that 74-78% of such transcripts were more abundant in *V. vinifera *and 22-26% were more abundant in *V. riparia*, depending on which time point was examined. When restricting the analysis to defense-related transcripts, the results were almost identical (74-76% vs. 24-26%) (Additional files 4 and 5). Overall, these data indicated that resistance in *V. riparia *does not reflect differences in the basal expression of defense-related genes.

### Transcriptional changes in *V. vinifera *and *V. riparia *in response to *P. viticola *infection

**Figure 2:**
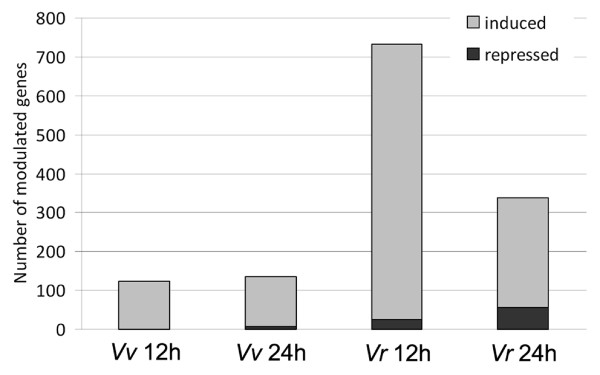
**Transcriptional changes associated with *P. viticola *infection**. Piled histograms represent the number of genes induced (gray bars) or repressed (black bars) in *V. vinifera *(*Vv*) and *V. riparia *(*Vr*), at 12 and 24 hpi with *P. viticola*.

Figure 2 shows the total number of transcripts that are differentially expressed (fold change ≥2) in the two species at 12 and 24 hpi (full list provided in Additional file 6). In both species, the majority of modulated transcripts were upregulated.

**Figure 3:**
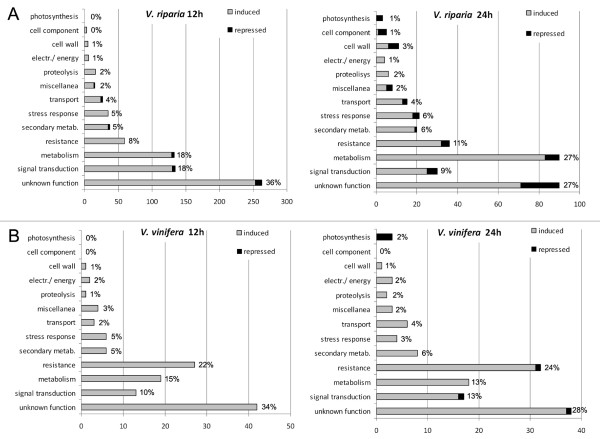
**Functional categories of transcripts modulated in *V riparia *and *V. vinifera *following infection with *P. viticola***. Transcripts modulated in *V. riparia *(**A**) and *V. vinifera *(**B**) after infection with *P. viticola *at 12 hpi (left panels) and 24 hpi (right panels) were manually grouped in functional categories on the basis of literature evaluation. Induced genes are represented in light gray, while repressed ones are in black. The total percentage of modulated transcripts within each category is shown next to each bar. The complete list of genes is available in Additional file 6.

*V. riparia *responded very quickly to infection, with 733 transcripts modulated at 12 hpi (707 induced, 26 repressed) whereas only 124 were modulated in *V. vinifera *(all induced) at the same time-point. At 24 hpi, 339 transcripts were modulated in *V. riparia *(283 induced, 56 repressed) whereas 135 were modulated in *V. vinifera *(129 induced, 6 repressed). The transcripts were assigned to functional categories on the basis of literature evaluation (Figure 3). Although the 'unknown function' category was predominant in both species, there were important differences in other categories. In *V. riparia*, signal transduction components accounted for 18% of the modulated transcripts at 12 hpi (almost invariably induced by infection) falling to 9% at 24 dpi, metabolic functions accounted for 18% of the modulated transcripts at 12 hpi increasing to 27% at 24 hpi, and defense-related functions accounted for 8% of the modulated transcripts at 12 hpi increasing slightly to 11% at 24 hpi. In *V. vinifera*, defense-related functions accounted for 22% of the modulated transcripts at 12 hpi increasing to 24% at 24 hpi, whereas metabolism and signal transduction accounted for 10-15% of modulated transcripts at both time-points. Other functional categories each accounted for up to 6% of modulated transcripts in both species at both time-points. Considering that a significant proportion of the differentially expressed genes are modulated at both time points, there were 870 differentially expressed transcripts in *V. riparia *and 187 in *V. vinifera*, with many modulated at both time points. Transcripts showing the greatest induction in response to infection (30-80-fold) tended to be induced in both species, albeit to different levels. They were predominantly defense-related transcripts and are discussed in more detail below.

### Common transcriptional changes in response to infection

**Figure 4:**
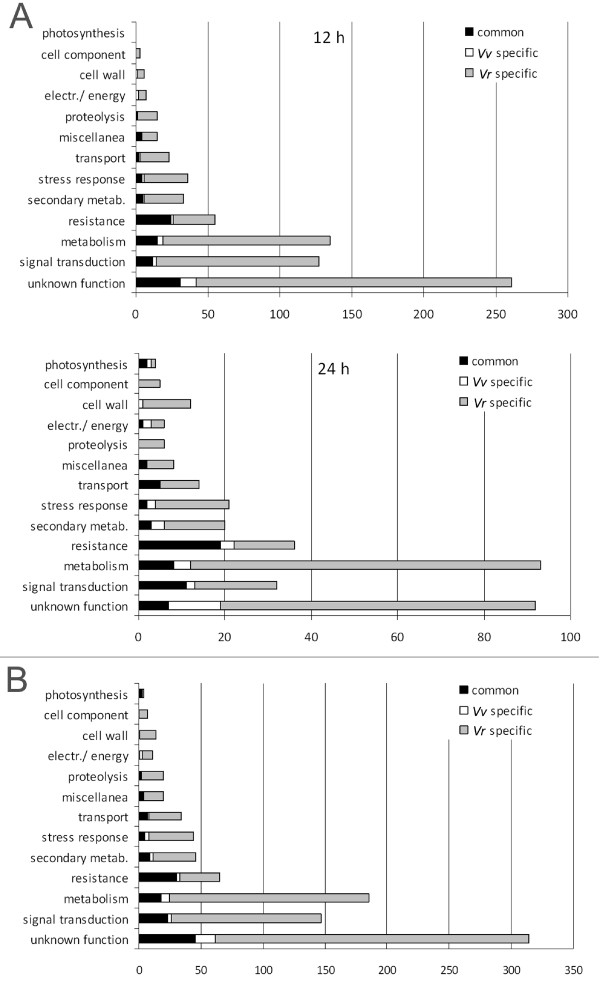
**Specificity of transcriptional changes in infected *V. vinifera *and *V. riparia *within selected functional categories**. **A**. Proportion of transcripts modulated in *V. vinifera *(*Vv*) or *V. riparia *(*Vr*) or in both species at either 12 (upper panel) or 24 hpi (lower panel). **B**. Proportion of transcripts modulated in *V. vinifera *(*Vv*) or *V. riparia *(*Vr*) or in both species considering either time point collectively.

Figure 4 shows the proportion of genes whose induction/repression in response to infection was observed in both species or was restricted to one or the other. This can be represented by a repartition of modulated transcripts by species, either at each time point (Figure 4A) or collectively (Figure 4B). We consider the second approach more useful because it defines modulations occurring in both species as common transcriptional changes, even though they may not occur at the same time. However, the first approach shows how specificity evolves over time, in some cases with different profiles in different functional categories.

**Figure 5:**
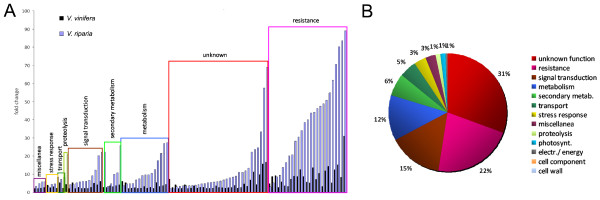
**Common transcriptional changes in *V. vinifera *and *V. riparia *following infection with *P. viticola***. **A**. Intensity of the transcriptional changes of 'common' genes in *V. riparia *and *V. vinifera *at 12 hpi. Each functional category is shown in a different color. **B**. Distribution of the 147 'common' genes, modulated in both species at one or both time points, into functional categories.

The data show clearly that most of the transcriptional modulation observed in *V. riparia *had no parallel in *V. vinifera*, indicating that many of the changes in all functional categories were restricted to *V. riparia*. In contrast, most of the transcriptional modulation observed in *V. vinifera *also occured in *V. riparia *(Figure 4A). However, when each species was considered separately at each time-point, it was clear that the number of transcripts uniquely modulated in *V. vinifera *increased from 12 to 24 hpi, possibly reflecting the establishment of a compatible interaction. Interestingly, the strength of modulation among the common genes was invariably much higher in *V. riparia*, at both 12 hpi (Figure 5A) and 24 hpi (data not shown).

When we considered as 'common' any gene that is modulated in both species irrespective of the time-point, we detected 147 common transcripts, always modulated in the same direction in both species (Figure 5B and Additional file 6). Moreover, because the timing of the response is also relevant, it is notable that 30% of the common transcripts were modulated earlier in *V. riparia *(12 hpi) and later in *V. vinifera *(24 hpi), although there is no qualitative difference associated with this delayed response (Additional file 6).

After discounting transcripts with no assigned function, the largest proportion of common transcripts were related to disease resistance (22%, Figure 5B). Within this category, about half of the transcripts modulated in *V. riparia *were also modulated in *V. vinifera*, including several encoding stilbene synthases and PR proteins such as chitinases, β-1,3-glucanases and PR-10. The difference in expression between the species was especially notable for these genes (Figure 5A). After resistance, the next largest group of common transcripts was related to signal transduction (15%, Figure 5B). This group included many transcripts encoding WRKY transcription factors, all strongly induced by infection at both time points, but again much more strongly induced in *V. riparia *(6-22-fold in *V. riparia*; 2-5-fold in *V. vinifera*). Approximately 12% of the common transcripts had metabolic functions, including a cell wall apoplastic invertase and an alternative oxidase, both of which were induced to a greater extent in *V. riparia*. Only a few genes related to photosynthesis were modulated in both species, and these were downregulated by infection (Additional file 7).

### Specific transcriptional changes in response to infection

Many transcriptional changes occurred solely in *V. riparia*, and the most prevalent functional categories among the modulated transcripts were general metabolism and signal transduction, the latter especially at 12 h. In the general metabolism category (22%; Figure 4B) most transcripts showed 2-3-fold induction, although a few were induced strongly, such as those encoding major enzymes in phenylalanine biosynthesis (up to 40-fold induction). Genes encoding enzymes in the Calvin cycle were repressed, in agreement with the decline in photosynthesis-related transcripts, whereas those involved in glycolysis and the pentose phosphate pathway were induced. Protein metabolism also appeared to be strongly influenced by infection, as shown by the large number of modulated transcripts related to ubiquitinylation, particularly those encoding different RING-H2 finger proteins, which are involved in proteolytic degradation (induced up to 14-fold). Transcriptional changes involving lipid metabolism included the upregulation of genes encoding biosynthetic and catabolic enzymes, and enzymes involved in jasmonic acid synthesis (e.g. allene oxide synthase and cyclase, omega-3 fatty acid desaturase). Several signal transduction pathways were affected including calcium signaling, ethylene signaling, MAP kinases, phosphatases, receptor-like proteins and numerous transcription factors. Overall, 68% of the signal transduction genes induced in *V. riparia *were never modulated in *V. vinifera*, and the vast majority were induced by 12 hpi (Figure 4; Additional file 6). Particularly strong modulation was observed for certain zinc-finger proteins (up to 16-fold induction) and WRKY genes (transient 3-4-fold induction) (Additional file 7).

We found that many resistance-related genes were induced to a greater or lesser extent in both species but those involved in the hypersensitive response were mostly restricted to *V. riparia*. These included several Avr9/Cf-9 rapidly elicited proteins [43] and a homolog of the tobacco *Hin1 *gene (12-fold induction) which is considered a HR marker [44]. Another HR marker, a homolog of the tomato *hsr203J *gene [45,46], was induced 40-fold in *V. riparia *and only 5-fold in *V. vinifera *at 12 hpi (Additional file 7).

There were few genes specifically induced in *V. vinifera *at 12 hpi, but the number increased substantially by 24 hpi. These genes represented several different functional categories and were not particularly informative with regard to the establishment of compatible interactions (Figure 4). Resistance and stress-related genes were well represented but on the whole it appeared that *V. vinifera *mounts a much less specific response to infection, which may be considered as an unsuccessful attempt to establish resistance.

### Validation of microarray analysis by real-time RT-PCR

The microarray data for 10 differentially expressed transcripts, whose induction index varied from 0.1-fold to 34-fold at either 12 or 24 hpi, were validated by real-time RT-PCR analysis. As shown in Additional file 8, the magnitude of change determined by the more sensitive real-time RT-PCR technique was in accordance with the microarray data and in some cases revealed even greater differential expression, suggesting that the microarray results underestimated actual changes in gene expression.

### Determination of jasmonate levels in infected leaves

**Figure 6:**
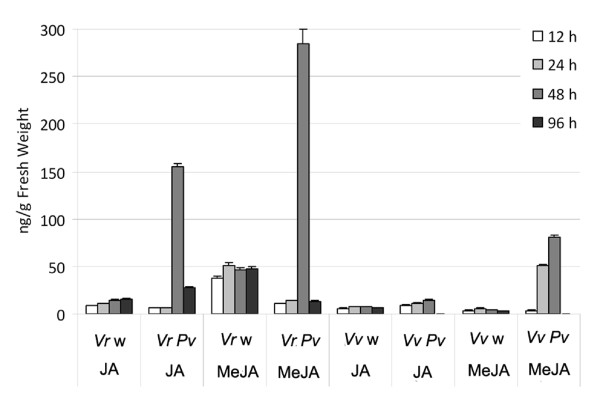
**Endogenous levels of jasmonic acid and MeJA in *V. riparia *(*Vr*) and *V. vinifera *(*Vv*)**. Measurements were taken using leaf samples collected at 12, 24, 28 and 96 hpi with *P. viticola *(*Pv*) or on the mock-inoculated control samples (w) at the corresponding time-points. Values are the average of three measurements, with standard errors.

The microarray data indicated that genes encoding enzymes involved in biosynthesis of jasmonic acid were strongly induced in *V. riparia *shortly after infection. We therefore measured the amount of jasmonic acid (JA) and methyl jasmonate (MeJA) in the leaves of both species before infection and at the four post-infection time-points discussed above. The basal levels of MeJA were higher in *V. riparia *than in *V. vinifera*. There was a sharp increase in the levels of both jasmonic acid and MeJA in *V. riparia *leaves 48 hpi, which was followed by rapid decline to below pre-infection levels (Figure 6). In *V. vinifera*, there was no change in the basal level of jasmonic acid after infection and only a limited increase in MeJA levels at 24 and 48 hpi.

## Discussion

### Analysis of *P. viticola *developmental stages

Infected tissues were examined under a microscope at 12, 24, 48 and 96 hpi, to determine the most suitable time-points for microarray analysis and to observe sporulation. The localization of zoospores over stomata at 12 hpi in both *V. riparia *and *V. vinifera *confirmed previous reports that zoospores can locate stomata with equal efficiency in susceptible and resistant species [27,30]. Restriction of pathogen growth in *V. riparia *is a post-infection phenomenon that begins when the first haustoria enter mesophyll cells, resulting in the thickening of cell walls, necrosis of guard cells, the accumulation of phenolics and peroxidases, and in some cases a hypersensitive reaction depending on environmental conditions [9,30,47]. This correlates well with the specific induction of genes related to hypersensitivity and phenylpropanoid synthesis. Pathogen spread was severely impaired between 24 and 48 hpi in comparison to *V. vinifera*, suggesting that the resistance mechanism is already in effect before this time point, consistent with the strong transcriptional reprogramming observed at 12 hpi, when the first haustoria form.

### Reliability of hybridization data

Because we used a *V. vinifera *microarray to assess differential gene expression in *V. vinifera *and *V. riparia *we performed experiments to confirm the reliability of cross-species hybridization. The successful outcome was not unexpected because *V. vinifera *arrays have previously been hybridized with RNA from other *Vitis *species [22,23,48]. Indeed, cross-species microarray hybridization is widely used in animals and plants [49-52], and although the data must be interpreted with caution, it remains a valid approach when dealing with groups of closely related species where sequence information is only available for one member [53]. The average signal intensity and the number of absent calls in the hybridization data were similar in *V. riparia *and *V. vinifera*, and comparison of replicates within each species suggested a similar level of variation. This probably indicates that polymorphisms within each species provide nearly as much sequence variation as the differences between species, as previously shown by singlenucleotide polymorphism analysis [54]. Moreover, the only direct comparison between *V. vinifera *and *V. riparia *was performed to assess differences in basal gene expression, while most of the comparisons were made between sampling time points in the same species, preventing such misinterpretation of hybridization results.

### Interspecies differences in basal gene expression

The comparison of basal gene expression in healthy *V. vinifera *and *V. riparia *plants 12 and 24 h after a mock infection procedure revealed substantial variation in the expression of thousands of genes, but no overall bias towards either species.

*V. riparia *is a major source of resistance against *P. viticola *[4,6,13,55,56] and although major resistance genes have been identified [8] it has been suggested that some resistance may be conferred by constitutive differences in defense-related gene expression. We therefore focused on defense-related transcripts (resistance, stress, cell wall and secondary metabolism categories) to see if there were any broad trends. Although the levels of individual transcripts varied widely, overall levels were similar in the two species (Additional file 3).

The 'cell wall' category contained more transcripts expressed preferentially in *V. riparia *and the average signal intensity was also higher, but the differential expression of various cell wall enzymes did not explain how the modified cell wall might help to prevent pathogen spread. The 'resistance' and 'stress' categories, in contrast, included more transcripts preferentially expressed in *V. vinifera*. Many grapevine species accumulate stilbene derivatives, such as resveratrols and viniferines, in response to pathogens [57,58] and we found that one stilbene synthase was preferentially expressed in *V. riparia *at 12 hpi, two were more abundant in *V. riparia *at 24 hpi, whereas five were more abundant in *V. vinifera*. Several PR protein genes were also more strongly expressed in *V. vinifera*, which is perhaps surprising because the genes are strongly induced by infection in *V. riparia *but not in *V. vinifera*. These data confirm that the response to *P. viticola *infection in *V. riparia *is not mediated by higher constitutive expression of defense genes and is essentially a post-infection process [26,28,30].

The absence of any significant differential expression of 'secondary metabolism' transcripts in pre-infection samples supports this conclusion, given that secondary metabolism, especially the phenypropanoid pathway, is often considered an important component of plant resistance [59]. In a previous microarray-based comparison of a susceptible and a resistant *V. vinifera *cultivars, Figueiredo and co-workers [21] identified only 12 genes preferentially expressed in the uninfected resistant cultivar, one of which encoded phenylalanine ammonia lyase, whereas 17 genes were preferentially expressed in the susceptible cultivar. Other authors have reported that stilbene synthase and phenylalanine ammonia lyase mRNA are not detected in healthy leaves but are induced by infection or abiotic stresses, proportionally to the resistance phenotype observed and are therefore considered elicitor-induced responses [24,25].

In the subset of transcripts showing differential basal expression at both time points, about 75% were more strongly expressed in *V. vinifera *and about 25% were more strongly expressed in *V. riparia*. When the analysis was restricted to defense-related transcripts the same broad trend was observed. Taken together, these findings suggest there is a stronger diurnal fluctuation in basal gene expression in *V. riparia *compared to *V. vinifera*, but provide no evidence that the resistance phenotype in *V. riparia *is caused by the constitutive expression of resistance genes maintaining a constant state of readiness.

### Broad transcriptional changes associated with *P. viticola *infection

The infection of both species with *P. viticola *results in the rapid induction of many genes, although their number and the magnitude of induction are much greater in *V. riparia *(Figure 3). Transcript profiling in other grapevine diseases [23,33,36,37] has focused on compatible interactions, for which large transcriptional changes are observed. The only incompatible interaction studied in this manner is that between *V. aestivalis *and the powdery mildew agent *Erysiphe necator *[23]. This is another biotrophic, haustoria-forming grapevine pathogen, which might be expected to adopt strategies similar to *P. viticola *with similar consequences. In *V. aestivalis *only three genes were shown to be modulated by infection by *E. necator*. The same authors also investigated the compatible interaction with *V. vinifera*, which responded with a broad remodeling of the transcriptome. Our data show that both *V. vinifera *and *V. riparia *respond to downy mildew infection with a massive transcriptional change, which is much more pronounced in the resistant species as suggested by several large scale analyses of incompatible interactions in other species [60-63]. Many similarities can be identified between the responses against powdery and downy mildew in *V. vinifera *based on the annotation of probes on the chips, although a complete and detailed comparison cannot be carried out because different array platforms were used in each case.

### Overlapping transcriptional responses to infection in *V. vinifera *and *V. riparia*

As expected, there were overlaps in the transcriptional changes in each species in response to infection, with most of the genes induced in *V. vinifera *constituting a weak subset of those induced in *V. riparia *at the same time-points (Figures 4 and 5). The limited response in *V. vinifera *appears to reflect an abortive attempt to achieve resistance, since most of the common modulated transcripts fall into the 'resistance' category (Figure 5). The activation of genes encoding PR proteins and enzymes in the phenylpropanoid pathway was anticipated based on data from model species [19,59]. Interestingly, many of the common modulated transcripts are not only expressed at higher levels in *V. riparia *than *V. vinifera*, but also at higher levels than the genes in the same family that are uniquely expressed in *V. riparia*, e.g. PR-10, stilbene synthases and WRKY transcription factors. For example, the six WRKY genes whose induction is common to both species (TC59548, TC66456, TC71038, TC57604, TC53734, TC68615) are induced 6-22-fold in *V. riparia*, whereas those solely expressed in *V. riparia *are induced 2-5-fold (TC60897, TC51831, TC51732, TC53072, TC55553, TC64282). It therefore appears that *V. vinifera *can only weakly execute those responses that are strongly induced in *V. riparia*.

It is interesting to highlight the induction of an apoplastic invertase (TC56057), a sink-specific enzyme that catalyzes the irreversible cleavage of sucrose into hexoses, both in *V. vinifera *and *V. riparia *(2-3-fold and 7-9-fold, respectively). The rapid induction of invertase activity has also been observed in tomato roots resistant to the necrotrophic fungal pathogen *Fusarium oxysporum *[64]. Likewise, in barley challenged with powdery mildew, an apoplastic invertase was induced more strongly and rapidly in a resistant cultivar [65]. Hexoses produced by the invertase could be seen as a nutrient source for pathogens, but also as a supply of extra energy required for the activation of defense responses [66,67] whose accumulation might suppress photosynthesis in line with our data on photosynthetic genes. Most importantly, sugar can also be used to trigger defense gene expression [68,69] hence the suggestion to consider apoplastic invertase as a true PR protein [66].

All the common genes were modulated in the same direction by both species, indicating they probably fulfill the same functions in defense. Inverse regulation of the same gene in genotypes with different infection outcomes could be interpreted as part of a pathogen defense suppression strategy [70]. Indeed, susceptibility to *P. viticola *is associated with broad downregulation of gene expression at later time-points [71] but our data show that such downregulation does not occur early in the infection.

Quantitative and kinetic differences between compatible and incompatible interactions have been elegantly described in Arabidopsis [61]. The incompatible interactions produced a more robust and intense transcriptional response and the proposed quantitative model suggested that a high level input signal is generated in resistant plants in response to infection, determining the robustness of the system.

### The specific transcriptional response in *V. riparia*

Although both species responded to infection with broad changes in gene expression, the response was strongest and fastest in *V. riparia*, with a peak of gene induction at 12 hpi. This response had transient and permanent components, since the expression of about half the genes fell back by 24 hpi (Figure 2). The strong transcriptional response of *V. riparia *together with its histological reactions to the pathogen is reminiscent of R-gene dependent resistance in other species [16], although the molecular determinants are unknown in this case.

When transcripts with unknown functions are excluded, the genes induced specifically in *V. riparia *fall into a number of functional categories whose expression appears to be coordinated. At 12 hpi, many genes encoding signal transduction components are induced, and this is followed by a wave of metabolic genes that are induced 24 hpi. This may indicate that an initial burst of signaling activity reprograms metabolism to provide a 'defense mode'. Among the different signaling pathways affected, calcium is known to be an important second messenger in resistance [72] as shown by the induction specifically in infected *V. riparia*, of calmodulins and calmodulin-binding proteins, calcium transporting ATPases, and proteins with similarity to calreticulin and calcineurin B-like proteins, all known to contribute to calcium homeostasis in the cell and to the definition of specific calcium signatures [73]. Several different ethylene response factors are also strongly induced solely in *V. riparia *at 12 hpi, and this hormone has also been implicated in resistance [74]. The possible involvement of ethylene in *P. viticola *resistance is further supported by the very strong induction of the ACC oxidase gene TC64623 (20-fold in *V. riparia *compared to only 3-fold in *V. vinifera*) and the 5-fold induction of an ACC synthase gene (TC60326) specifically in *V. riparia*.

Several genes with homology to known receptor-like protein kinases and leucine-rich repeat receptor-like proteins are specifically induced in *V. riparia*, especially at 12 hpi. These genes are known to mediate pathogen recognition and trigger defense responses in many species [75]. Although the ligands for these receptors are unknown, hundreds of genes encoding receptor-like proteins have been identified in *V. vinifera *[12,13], some of which map in linkage groups associated with resistance. Two MAP kinase kinase genes (TC62930, TC53469) were induced specifically in *V. riparia *at 12 hpi, consistent with the upregulation of three MAP kinases, two specifically in *V. riparia *at 12 hpi (TC66292, TC56256) and one also induced in *V. vinifera *at 24 hpi (TC61436). Interestingly, the TC66292 and TC56256 genes are related to Arabidopsis MAP kinase 3 (MPK3), the ortholog of tobacco wound-induced protein kinase (WIPK), which acts together with salicylic acid-induced protein kinase (SIPK) in resistance responses [76]. The absence of a SIPK homolog among our induced genes is consistent with its predominantly post-translational mode of regulation [77].

Several families of transcription factors are also specifically upregulated in *V. riparia*, especially WRKY factors and other zinc-finger proteins. WRKY factors are regulated by interaction with MAP kinase in other species [78,79] which provides a link in the signaling network we have outlined above. WRKY factors bind to DNA motifs known as W-boxes which are often found in defense genes, so they are regarded as important regulators of resistance [80].

It is well established that primary metabolic reprogramming underlies defense in biotrophic interactions and many genes in this category are specifically induced in *V. riparia*. Further analysis of our data suggests that specific pathways are involved: gycolysis (GADPH, enolase), the pentose phosphate pathway (glucose 6-phosphate dehydrogenase) and the Krebs cycle (pyruvate dehydrogenase, citrate synthases, succinyl-CoA ligase) are all induced, and could supply both energy and precursors for the biosynthesis of aromatic amino acids. Indeed, we observed the strong and specific induction of a group of genes controlling all the key steps in phenylalanine biosynthesis, including genes with homology to 3-deoxy-D-arabino-heptulosonate 7-phosphate synthases (6-30 fold at 12 hpi), chorismate synthase and mutase, and prephenate dehydratase, correlating with the induction of PAL (GSVIVT00013936001) and other genes involved in the hydroxycinnamic acid biosynthesis. Enzymes involved in lipid metabolism are also induced specifically in *V. riparia*. These include enzymes involved in lipid synthesis (e.g. acetyl-CoA carboxylase, β-ketoacyl-CoA synthase) and degradation (e.g. 13-lipoxygenase, acyl-CoA oxidase, acetoacetyl-CoA thiolase), and enzymes involved in the synthesis of jasmonates (omega-3 fatty acid desaturase, allene oxide cyclase, allene oxide synthase).

Genes encoding anti-oxidant enzymes and genes involved in protein degradation are also strongly and specifically induced in *V. riparia*, e.g. many RING-H2 domain proteins involved in ubiquitinylation are induced at 12 hpi. Interestingly, a rice RING-H2 protein associated with incompatible (but not compatible) interactions with *Magnaporthe grisea *is induced following treatment with different resistance-inducing chemicals, and transgenic plants constitutively expressing this gene are resistant to several pathogens, as well as drought and oxidative stress [81]. This demonstrates how modulated transcripts identified in our experiments provide promising candidates for biotechnology-based disease resistance programs.

Surprisingly, 'resistance' as a functional category, is relatively poorly represented among genes expressed specifically in *V. riparia*, many of them instead being common to both species. However, as already stated, many of the common resistance genes are more strongly modulated in *V. riparia*, and the *V. riparia*-specific group does include a number of genes strictly related to hypersensitivity, such as those encoding rapidly elicited Avr9/Cf-9 proteins (e.g. TC63609, TC61603) [43], two hypersensitive-induced response proteins (TC63023, TC63883) and two homologs of known HR markers in other species - tobacco *Hin1 *[44] and tomato *hsr203J *[45,46] - both of which are specifically or preferentially induced in *V. riparia *at 12 hpi. The HR has previously been implicated in resistance response to downy mildew in *V. riparia *[27]. Several additional defense genes are strongly induced in *V. riparia*, including those encoding PR proteins (such PR-4 and PR-10) and enzymes involved in the synthesis of antimicrobial compounds, as already reported in grapevine infected with powdery and downy mildew [23,28].

### The specific transcriptional response in *V. vinifera*

Although most modulated transcripts in *V. vinifera *are also modulated in *V. riparia*, there is a small collection of genes induced specifically in *V. vinifera*. The genes involved in this specific response do not suggest any coordinated and explicit mechanism related to the establishment of compatibility in *V. vinifera*. It is possible that the analysis of early transcriptional changes provides more information on resistance than susceptibility (the former involving a pro-active transcriptional response by the plant) and transcriptional changes associated with compatibility are established later [71].

### Jasmonate levels in healthy and infected plants

Resistance to biotrophic pathogens is often dependent on salicylic acid-mediated defense responses [82]. Jasmonates were originally associated with defense against herbivores and necrotrophic pathogens [83] but have more recently been implicated in resistance against biotrophes, such as powdery and downy mildews in Arabidopsis and in grapevine [84-87] and in resistance induced by BABA and by β-1,3-glucan sulfate against *P. viticola *[88,89]. Jasmonates interact with other danger signals such as salicylic acid and ethylene to determine the ultimate outcome of an infection, in a manner dependent on the specific plant-microbe interaction. Our data support a role for jasmonates in establishing or maintaining *V. riparia *resistance against *P. viticola*, given the significant increase in the levels of both jasmonic acid and MeJA at 48 hpi only in this species, concomitant with the effective arrest of pathogen growth, although much later in comparison to the transcriptional reprogramming described above. More experiments are needed to determine the precise timing of this accumulation in relation to pathogen arrest and to reveal how much of the response to *P. viticola *can be considered jasmonate-dependent in grapevine.

## Conclusions

We compared two grapevine species, *V. riparia *and *V. vinifera*, the former resistant to the pathogen *P. viticola *and the latter susceptible to infection. Comparative transcriptome analysis of healthy leaves and leaves representing two early infection stages allowed us to characterize the molecular events involved in the establishment of resistance in *Vitis riparia*.

Our data strongly support the view that resistance in *Vitis riparia *is a post-infection phenomenon, characterized by a rapid wave of signal transduction (12 hpi) followed by a shift in primary and secondary metabolism (24 hpi) to implement a defense mode. In contrast, early transcriptional changes in *V. vinifera *indicate a weak and abortive defense response and do not provide information about the possible downregulation of resistance mechanisms by pathogen effectors, which might occur later on. Basal levels of defense gene expression in the two species do not seem to be responsible for the different infection outcomes.

The upregulation of genes involved in jasmonic acid biosynthesis and the increase in jasmonate levels indicate that this hormone may play a role in *V. riparia *resistance against *P. viticola*, although signal transduction-related genes are already upregulated before a detectable increase of jasmonate accumulation. Our broad comparative characterization of resistant and susceptible phenotypes has provided several candidate genes that could be used for additional functional analysis and for the development of disease-resistant commercial grapevine varieties in the future.

## Methods

### Plant material and *P. viticola *infections

*Vitis vinifera *cv. Pinot Noir and *Vitis riparia *cv. Gloire de Montpellier plants were grown *in vitro *at 27°C with a 16-h photoperiod (50 μE/m2/s) as described by Blaich [90]. The *P. viticola *isolate was harvested in experimental fields in 2007 and propagated axenically on surface-sterilized detached Pinot Noir leaves maintained in Petri dishes. Five days after inoculation, sporangia were collected from freshly-sporulating leaves using a microtip equipped with a nylon filter and connected to a vacuum pump. In order to obtain uncontaminated sporangia, the inoculum was repeatedly propagated under axenic conditions on plants growing *in vitro*.

Fully expanded leaves of 8-10-week-old *in vitro *plants were infected by applying 50-μl drops containing 50,000 sporangia per ml on the adaxial leaf surface (or distilled water as a control). The concentration of sporangia was determined using a hemocytometer. For microscopy, leaf disks were collected 12, 24, 48 and 96 hpi, stained with 0.05% (w/v) aniline blue in 0.1% (w/v) Na_2_CO_3 _(pH 10), and observed under an epifluorescence microscope (Leica DM/RB, excitation filter BP 340-380 nm; dichroic mirror 400 nm; suppression filter LP > 430 nm). For microarray analysis, leaf disks were collected 12 and 24 hpi, immediately frozen in liquid nitrogen and stored at -80°C. Three independent biological replicates of the artificial infection were performed.

### Combimatrix array conception

The analysis was performed on a Combimatrix *Vitis vinifera *chip produced by the Plant Functional Genomics Center at the University of Verona. The chip contained 24,571 non-redundant probes in triplicate, composed of 35-40-mer oligos. Probes were designed using the program oligoarray 2.1 [91] and were based on tentative consensus sequences (TCs) derived from the TIGR *Vitis vinifera *Gene Index release 5.0 (19062 probes), singletons with a 3' poly(A) tail (1904 probes), expressed sequence tags (55 probes) and on genomic sequences produced by the International Grape Genome Project [11] that were not already represented by the TCs (3490 probes). TC annotations were derived from the TIGR Gene Index, release 5.0 and EST annotations were obtained by aligning sequences against UniProtKB/Swiss-Prot database with BLASTX. Nine bacterial oligonucleotide sequences provided by CombiMatrix, 40 probes designed on seven Ambion spikes and 11 additional negative probes based on *Bacillus anthracis*, *Haemophilus ducreyi *and *Alteromonas phage *sequences were used as negative controls. Three or four replicates of each probe were distributed randomly across the array. Two technical and three biological replicates were used for each hybridization experiment.

### RNA preparation, hybridization and microarray analysis

RNA was isolated according to Reid et al. (2006) and quantified by spectrophotometry (ATI Unicam) and using an Agilent 2100 Bioanalyzer. Total RNA (1 μg) was amplified using the SuperScript Indirect RNA Amplification System (Invitrogen, USA), to incorporate amino-allyl UTP molecules (aRNA) and a fluorescent label (Alexa Fluor 647). The purified, labeled aRNA was quantified by spectrophotometry and 4 μg was hybridized to the Combimatrix array according to the manufacturer's directions. Pre-hybridization, hybridization, washing and imaging were performed according to the manufacturer's protocols http://www.combimatrix.com/support_docs.htm. The array was scanned with a ScanArray 4000XL (Perkin-Elmer, USA) and TIF images were exported to Microarray Imager 5.8 (CombiMatrix, USA) for densitometric analysis. Probe signals higher than negative control values plus twice the standard deviation were considered as 'present'. Data were normalized by quantile normalization and differentially expressed genes were identified using the Two Class Unpaired Statistical Analysis of Microarrays method [92] with a False Discovery Rate (FDR) < 5%. Expression data are available from the National Center for Biotechnology Information (NCBI) [GenBank: Gene Expression Omnibus accession number GSE18596].

### Real-Time RT-PCR

Real-Time RT-PCR experiments were carried out in biological triplicates with the same RNA samples taken for microarray analysis, using the SYBR^® ^Green PCR master mix (Applied Biosystems, Foster City, CA, USA) and the Mx3000P Real-Time PCR System (Stratagene, La Jolla, CA, USA). Complementary DNA was synthesized from DNase-treated total RNA using the ImProm-II Reverse Transcription System (Promega, Madison, WI, USA). Gene-specific primers were designed for the 10 target genes as well as the actin transcript TC81781 (see Additional file 9). Each 25-μl reaction comprised 300 nM each primer and cDNA synthesized from 40 ng of total RNA (three replicates for each reaction) and began with a 50°C hold for 2 min and a 95°C hold for 10 min followed by 40 cycles at 95°C for 30 s, 55°C for 30 s, and 72°C for 20 s. Non-specific PCR products were identified by analyzing dissociation curves. The amplification efficiency was calculated from raw data using LinRegPCR software [93]. The relative expression ratio value was calculated for treated samples relative to the corresponding untreated sample at the same time-point according to the Pfaffl equation [94]. SE values were calculated according to Pfaffl et al. [95].

### Analysis of endogenous jasmonic acid and methyl jasmonate levels

Frozen plant material (500 mg fresh weight) was pulverized under liquid nitrogen, mixed with 4 ml methanol and filtered into a vial. After repeating this procedure twice, the extract was divided into two aliquots and the solvent evaporated under nitrogen at room temperature. To estimate the jasmonic acid content, 2 ml of ethereal trimethylsilyldiazomethane (2M in diethyl ether, Sigma-Aldrich) was added to the dried sample and incubated for 30 min before stopping the reaction under a gentle stream of nitrogen. The dried sample was mixed with 1 ml 30% NaCl and methylated jasmonic acid was extracted by solid phase micro-extraction (PDMS 100 μm film thickness, Supelco) while stirring at 60°C for 30 min. Blank analyses were carried using saline. Preliminary recovery studies were performed by adding known amounts of jasmonic acid (5, 10, 50, 100 and 200 ng) to grapevine leaf tissue prior to extraction, with recovery in the range 85-93%.

To estimate the levels of endogenous MeJA, plant material was extracted by solid phase micro-extraction without the derivatization step. The amount of endogenous MeJA was then subtracted from the total methylated jasmonic acid level to calculate the concentration of JA in the samples [96]. The limit of detection for jasmonic acid as MeJA was 2 ng/g.

GC-analysis was performed with a Varian CP-3800 (Varian Inc., Palo Alto, CA, USA) equipped with a 1177 split/splitless injector, a Factor-Four 5 capillary column (Varian 30 m, ID 0.25 mm, F.t. 0.25 μm), a FID detector and a Galaxie Workstation software (Varian Inc.) [96]. GC-MS analyses were also used to confirm the efficacy of the methylation procedure with a Varian Saturn 2100 GC-MS operating in the electron impact mode (EI), equipped with a multiple-ion detector and a Factor-Four 5 capillary column (Varian 30 m, ID 0.25 mm, F.t. 0.25 μm) as described [96].

## Authors' contributions

MP performed grapevine infections, microscopic examinations, RNA extractions and microarray hybridizations. LB helped writing the manuscript and prepared all Figures and Additional files. AF collaborated in designing the Combimatrix grapevine gene chip, in fluorescent data extraction, and performed microarray statistical analysis. AZ collaborated in statistical analysis of microarray and real time RT-PCR data. MF performed real time RT-PCR experiments. CZ performed jasmonic acid and methyl jasmonate measurements. AL was responsible for growing *in vitro *plants and for *P. viticola *maintenance and collaborated to infection experiments. MPz and MD hold the responsibility of the Plant Functional Genomic Centre that produced the Combimatrix grapevine gene chip and collaborated to experimental design. AP conceived the study, participated in all steps of the analysis and wrote the manuscript. All authors read and approved the final manuscript.

## Supplementary Material

Additional file 1 **Differences in basal gene expression levels between the two species at 12 h after mock-inoculation with distilled water**. The file contains a list of transcripts showing statistically significant differential expression, with a False Discovery Rate (FDR) ≤5%. The fold change of *V. vinifera *vs. *V. riparia *expression levels (Fold Change *Vv*/*Vr*) is reported, along with the q-value (%) indicating the FDR. A separate list reports the subset of defense-related genes, functionally categorized as 'resistance', 'stress', 'cell wall' and 'secondary metabolism' considered in Additional file 3. Click here for file

Additional file 2 **Differences in basal gene expression levels between the two species at 24 h after mock-inoculation with distilled water**. The file contains a list of transcripts showing statistically significant differential expression, with a False Discovery Rate (FDR) ≤5%. The fold change of *V. vinifera *vs. *V. riparia *expression levels (Fold Change *Vv*/*Vr*) is reported, along with the q-value (%) indicating the FDR. A separate list reports the subset of defense-related genes, functionally categorized as 'resistance', 'stress', 'cell wall' and 'secondary metabolism' considered in Additional file 3. Click here for file

Additional file 3 **Comparison between defense-related genes in *V. vinifera *and *V. riparia *at 12 and 24 h after mock-inoculation with distilled water**. Defense-related genes considered for the comparison are those functionally categorized as 'resistance', 'stress', 'cell wall' and 'secondary metabolism' and are shown in the 'defense-related' lists in Additional files 1 and 2. The tables on the left show the total numbers of genes whose basal expression is higher in *V. riparia *(overexpressed in *Vr*) or *V. vinifera *(overexpressed in *Vv*) within each category. The tables on the right report mean logarithmic fluorescence values of transcripts within each category (mean *Vr *and mean *Vv*), the ratio of the means calculated for each genotype and the resulting fold change. Microarray fluorescence data from the two time-points were normalized and analyzed separately to avoid detecting basal differences based on the response to illumination. Click here for file

Additional file 4 **Subset of transcripts showing a difference in basal expression level between *V. vinifera *and *V. riparia *at both the 12 and 24 h time points after mock-inoculation with distilled water**. The file contains a list of transcripts showing statistically significant differential expression, with a False Discovery Rate (FDR) ≤5%. The fold change of *V. vinifera *vs. *V. riparia *expression levels (Fold Change *Vv*/*Vr*) is reported, along with the q-value (%) indicating the FDR. A separate list reports the subset of defense-related genes, functionally categorized as 'resistance', 'stress', 'cell wall' and 'secondary metabolism' considered in Additional file 5. Click here for file

Additional file 5 **Comparison between defense-related genes in the subset of transcripts differentially expressed in the two species both at 12 and 24 h after mock-inoculation with distilled water**. Defense-related genes considered for the comparison are those functionally categorized as 'resistance', 'stress', 'cell wall' and 'secondary metabolism' and are shown in the 'defense-related' list in Additional file 4. The tables on the left show the total numbers of genes whose basal expression is higher in *V. riparia *(overexpressed in *Vr*) or *V. vinifera *(overexpressed in *Vv*) within each category. The tables on the right report mean logarithmic fluorescence values of transcripts within each category (mean *Vr *and mean *Vv*), the ratio of the means calculated for each genotype and the resulting fold change. Click here for file

Additional file 6 **Differential gene expression in *V. riparia *and *V. vinifera *following infection with *P. viticola***. The file lists transcripts showing a statistically significant differential expression (fold change ≥2, FDR ≤5%) in *P. viticola *infected samples of *V. riparia *(*Vr*) and *V. vinifera *(*Vv*), in comparison to their respective mock-inoculated controls, at 12 and 24 hpi. Species-specific and 'common' transcriptional changes associated with infection are also reported in separate lists for easier access. Click here for file

Additional file 7 **Representative *V. riparia *and *V. vinifera *transcripts modulated after infection with *P. viticola***. A selection of representative transcripts modulated in both species ('common') or specifically in *V. riparia *(*Vr*) or *V. vinifera *(*Vv*) after infection with *P. viticola*. Target descriptions are provided, corresponding to gene annotations in the source databases, along with the corresponding functional category and the microarray fold change (FC) value for each time point. Click here for file

Additional file 8 **Real-Time RT-PCR analysis of selected genes**. The figure reports the comparison of transcriptional changes of selected genes as determined by microarray (white bars) and Real-Time RT-PCR analysis (black bars). The black bars indicate the average fold change obtained for the three independent biological replicates, and the error bars indicate the standard deviations. Individual fold change values and standard errors for each Real-Time experiment are available in Additional file 9. Click here for file

Additional file 9 **Details of the Real-Time RT-PCR analysis**. The file contains: the sequence ID of each gene analyzed by Real-Time RT-PCR; the corresponding primer pairs used for the amplification (FOR = forward primer, REV = reverse primer); an indication of the region amplified by each primer pair (3' UTR = 3'untranslated region; CDS = coding sequence; CDS-probe = region of the coding sequence covered by the microarray probe; CDS-3' UTR = region between the coding sequence and the 3'untranslated region); the time point after treatment at which the leaves were sampled; the Real-Time RT-PCR results, reported as fold change (FC) relative to the untreated control sample and with the standard error (SE), for each species (*Vv *= *V. vinifera Vr *= *V. riparia*) and divided in biological replicates (1, 2 and 3); the mean values of the FC for the three replicates for each genotype. The corresponding microarray results for the same transcripts are also reported as fold change at the end of the list. Click here for file
