# Supplementary figures and images for: General and species-specific transcriptional responses to downy mildew infection in a susceptible (Vitis vinifera) and a resistant (V. riparia) grapevine species
Source: BMC Genomics. 2010 Feb 18;11:117. doi: 10.1186/1471-2164-11-117 (PMC2831845; doi:10.1186/1471-2164-11-117)

# *V. vinifera*

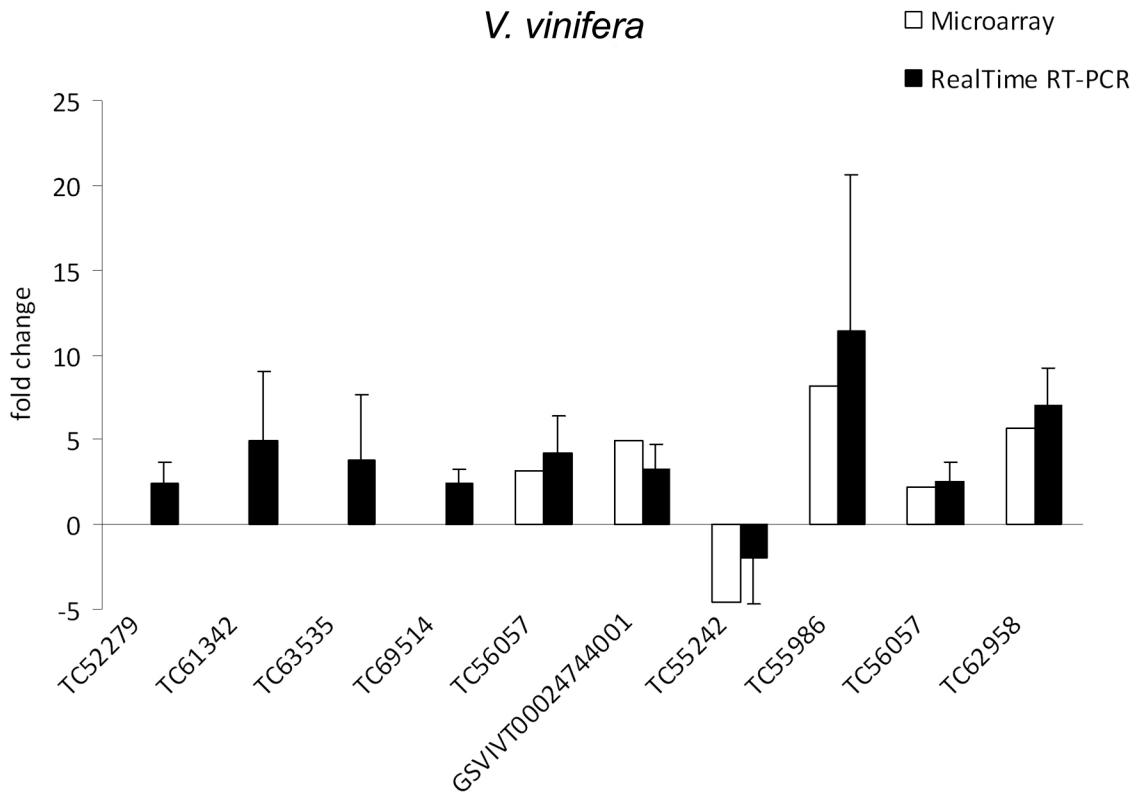

# *V. riparia*

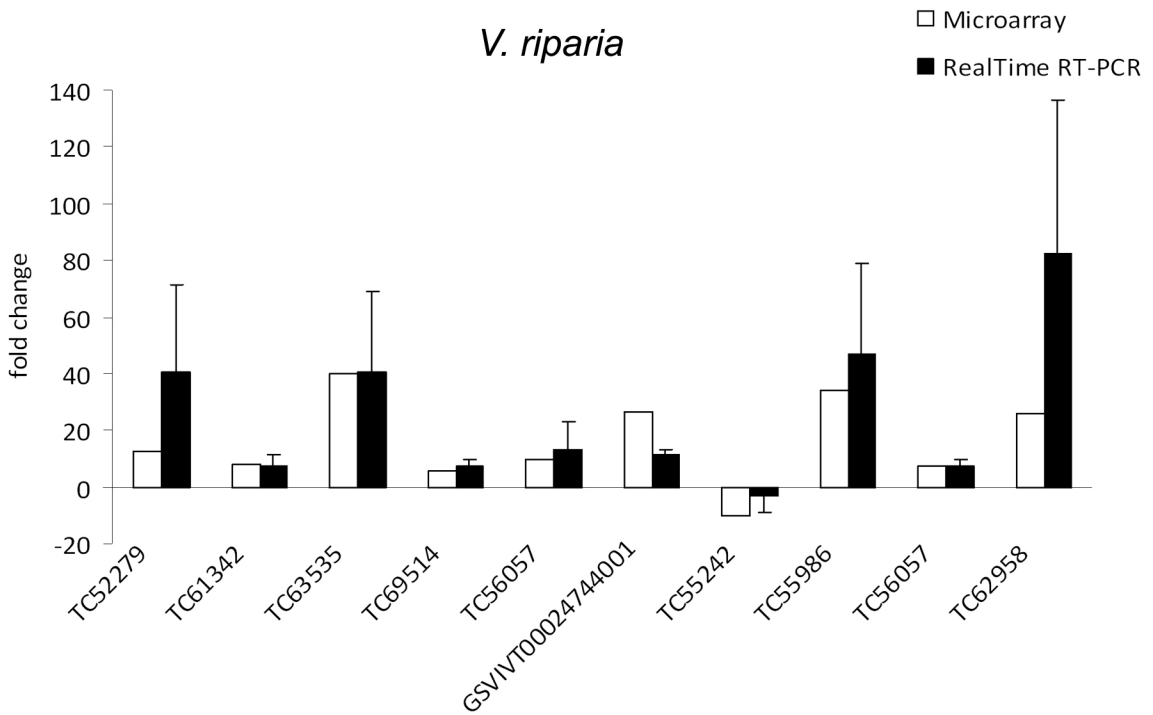

Supplement: Additional file 8 — Real-Time RT-PCR analysis of selected genes. The figure reports the comparison of transcriptional changes of selected genes as determined by microarray (white bars) and Real-Time RT-PCR analysis (black bars). The black bars indicate the average fold change obtained for the three independent biological replicates, and the error bars indicate the standard deviations. Individual fold change values and standard errors for each Real-Time experiment are available in Additional file 9. [file 1471-2164-11-117-S8.PDF]
